# Neuron-Specific Enolase for Neurological Prognostication After Cardiac Arrest in the Era of Targeted Temperature Management: A Systematic Review and Meta-Analysis

**DOI:** 10.3390/jcm15155798

**Published:** 2026-07-24

**Authors:** Raluca Maria Badila, Alina Simona Bereanu, Mihai Sava, Sandra Ioana Neamtu, Bogdan Ioan Vintila, Ioana Roxana Codru, Corina Roman Filip

**Affiliations:** 1 County Clinical Emergency Hospital of Sibiu, 550245 Sibiu, Romania; mihai.sava@ulbsibiu.ro (M.S.); bogdan.vintila@ulbsibiu.ro (B.I.V.);; 2 Faculty of Medicine, Lucian Blaga University of Sibiu, 550024 Sibiu, Romania

**Keywords:** cardiac arrest, neuron-specific enolase, targeted temperature management, therapeutic hypothermia, neuroprognostication, biomarkers, post-cardiac arrest syndrome, systematic review, meta-analysis

## Abstract

**Background/Objectives**: Neuron-specific enolase (NSE) is one of the most extensively investigated biomarkers for neurological prognostication after cardiac arrest. However, uncertainty remains regarding the influence of targeted temperature management (TTM) on NSE kinetics, optimal prognostic thresholds, and predictive accuracy. This systematic review and meta-analysis aimed to evaluate the prognostic performance of NSE and assess the impact of TTM on its clinical utility in contemporary post-resuscitation care. **Methods**: A systematic search of PubMed/MEDLINE, Embase, and Scopus was conducted to identify studies published between January 2000 and April 2026. Literature screening, data extraction, and quality assessment were performed in accordance with the PRISMA 2020 statement. Methodological quality was assessed using the Newcastle–Ottawa Scale and the Cochrane Risk of Bias tool, when applicable. Quantitative synthesis was performed for studies reporting sufficiently homogeneous area under the receiver operating characteristic curve (AUC) data. **Results**: Twenty-one studies met the eligibility criteria and were included in the qualitative synthesis, of which seven were eligible for quantitative analysis. NSE demonstrated good overall prognostic performance for predicting neurological outcome after cardiac arrest, with a pooled AUC of 0.868 (95% CI: 0.849–0.885) and no significant between-study heterogeneity (I^2^ = 0%). Prognostic performance was generally highest when NSE was measured between 48 and 72 h after return of spontaneous circulation. Considerable variability was observed in reported prognostic thresholds, particularly across different TTM strategies. Serial NSE measurements and multimodal neuroprognostication approaches consistently improved predictive performance. **Conclusions**: NSE provides good prognostic accuracy for neurological outcome prediction after cardiac arrest and remains clinically valuable in patients managed with TTM. Serial measurements obtained between 48 and 72 h after return of spontaneous circulation appear to provide the greatest prognostic value. NSE should be interpreted as part of a multimodal neuroprognostication strategy rather than as a standalone predictor.

## 1. Introduction

Cardiac arrest remains a major cause of mortality and long-term neurological disability worldwide despite substantial advances in cardiopulmonary resuscitation and post-resuscitation critical care [1,2]. Although return of spontaneous circulation (ROSC) can frequently be achieved, hypoxic–ischemic brain injury continues to represent the principal determinant of survival and neurological recovery [3]. Consequently, early and reliable neuroprognostication has become a central component of contemporary post-cardiac arrest management, directly influencing therapeutic decisions and withdrawal of life-sustaining treatment [4].

Current international guidelines advocate a multimodal neuroprognostication strategy integrating clinical examination, electrophysiology, neuroimaging, and circulating biomarkers in order to improve prognostic accuracy and minimize false pessimistic predictions [5]. However, reliable prognostication remains particularly challenging in patients undergoing targeted temperature management (TTM), as sedation, delayed awakening, and metabolic disturbances may interfere with neurological assessment [6]. In this context, blood biomarkers have emerged as valuable adjunctive tools for evaluating the severity of hypoxic–ischemic brain injury and refining prognostic evaluation.

Among currently available biomarkers, neuron-specific enolase (NSE) remains the most extensively investigated and clinically implemented serum marker after cardiac arrest [7]. NSE is a glycolytic isoenzyme localized predominantly within neurons and neuroendocrine cells and released into the circulation following neuronal membrane disruption and hypoxic–ischemic injury. Elevated serum NSE concentrations have consistently been associated with poor neurological outcomes and increased mortality in both observational cohorts and randomized post-cardiac arrest studies [7]. Consequently, serial NSE measurements obtained at 48–72 h after ROSC have been incorporated into contemporary ERC/ESICM neuroprognostication algorithms [5].

Despite its widespread clinical use, the interpretation of NSE remains controversial. Significant heterogeneity persists regarding optimal cutoff thresholds, timing of biomarker sampling, assay methodologies, and definitions of unfavorable neurological outcome across published studies [8]. Moreover, several confounding factors—including hemolysis, extracerebral NSE release, renal dysfunction, extracorporeal support therapies, and laboratory variability—may influence circulating NSE concentrations and reduce prognostic specificity [9]. Recent evidence has demonstrated that falsely elevated NSE values may occur even in patients with favorable neurological recovery, emphasizing the limitations of isolated threshold-based interpretation and supporting the need for multimodal prognostic integration [9,10].

Targeted temperature management represents another important challenge in NSE interpretation. Contemporary targeted temperature management strategies remain central to post-resuscitation care after cardiac arrest, although recent evidence has increasingly emphasized individualized temperature targets and multimodal neuroprognostication approaches [11,12]. Through modulation of cerebral metabolism, inflammatory cascades, oxidative stress, and delayed neuronal injury, TTM may also alter biomarker kinetics and influence the prognostic performance of circulating biomarkers [13]. Consequently, uncertainty persists regarding whether NSE thresholds established in earlier studies remain applicable under contemporary temperature management strategies.

During recent years, substantial attention has focused on NSE interpretation in the era of TTM and multimodal neuroprognostication. The TTM2 trial challenged previous assumptions regarding the superiority of hypothermia over active normothermia and renewed interest in biomarker-guided prognostic assessment under different temperature management strategies [14]. Simultaneously, contemporary investigations published between 2024 and 2026 have significantly expanded the current understanding of NSE-based prognostication.

Kim et al. demonstrated that even “normal-range” NSE concentrations may possess predictive utility for favorable neurological recovery after out-of-hospital cardiac arrest, suggesting that low biomarker values may carry important prognostic significance [10]. Pelle et al. subsequently reported that individualized NSE interpretation according to electroencephalographic patterns improved prognostic discrimination and reduced false-positive predictions, reinforcing the importance of integrating biomarkers into multimodal prognostic frameworks [15]. Additional studies have focused on methodological limitations influencing NSE reliability, including hemolysis correction strategies, assay standardization, and serial biomarker kinetics [16,17,18].

One of the most important recent contributions to the field is the international prospective biomarker substudy conducted within the Targeted Hypothermia versus Normothermia after Out-of-Hospital Cardiac Arrest (TTM2) trial [19]. In this large multicenter investigation, Moseby-Knappe et al. evaluated multiple circulating biomarkers of brain injury after cardiac arrest and demonstrated substantial differences in prognostic performance among available biomarkers [19]. Their findings reinforced the continuing clinical relevance of NSE while simultaneously emphasizing persistent uncertainty regarding optimal thresholds and the need for improved multimodal neuroprognostication strategies [19]. Similarly, recent studies evaluating multimodal neuroprognostication integrating serum biomarkers, electroencephalography, and neuroimaging have suggested superior predictive performance compared with isolated biomarker assessment alone [15,18].

Although several systematic reviews and meta-analyses have previously evaluated NSE after cardiac arrest, many were conducted before publication of recent TTM studies, updated international guidelines, and contemporary biomarker investigations addressing individualized thresholds, assay variability, and multimodal prognostic integration [8]. Furthermore, evidence remains fragmented regarding the specific influence of TTM strategies on NSE kinetics, prognostic thresholds, and predictive accuracy. Given the increasing clinical reliance on biomarker-guided neuroprognostication, an updated synthesis of the available evidence incorporating contemporary TTM-era studies is warranted.

Accordingly, this systematic review and meta-analysis aims to evaluate the prognostic accuracy of serum neuron-specific enolase after cardiac arrest and to investigate the impact of targeted temperature management on NSE kinetics, threshold interpretation, and prognostic performance.

## 2. Materials and Methods

### 2.1. Literature Search Strategy

A systematic literature search was performed using the PubMed/MEDLINE, Embase, and Scopus databases to identify studies evaluating neuron-specific enolase (NSE) after cardiac arrest. The search strategy combined Medical Subject Headings (MeSH) terms and free-text keywords related to cardiac arrest, targeted temperature management, neuroprognostication, and neuron-specific enolase.

The following search terms were used in different combinations: “cardiac arrest”, “out-of-hospital cardiac arrest”, “in-hospital cardiac arrest”, “post-cardiac arrest syndrome”, “neuron-specific enolase”, “NSE”, “targeted temperature management”, “therapeutic hypothermia”, “normothermia”, “neuroprognostication”, “biomarkers”, and “hypoxic-ischemic brain injury”.

The literature search included studies published from January 2000 to April 2026, and the final search update was performed on 30 April 2026. Additional studies were identified through manual screening of reference lists from eligible articles and relevant review papers. The complete database-specific search strategies, including MeSH terms, Boolean operators, search restrictions, and database syntax, are provided in Supplementary Material S2.

This systematic review and meta-analysis were conducted in accordance with the Preferred Reporting Items for Systematic Reviews and Meta-Analyses (PRISMA 2020) statement [20]. The completed PRISMA checklist is provided in the Supplementary Materials. The study protocol was prospectively registered in the International Prospective Register of Systematic Reviews (PROSPERO; registration number CRD420261389234).

Ethical approval was not required because this study was based exclusively on previously published data and did not involve direct patient participation.

### 2.2. Inclusion and Exclusion Criteria

The inclusion and exclusion criteria were predefined according to the PECO/PICOS framework.

The inclusion criteria were as follows: (1) randomized controlled trials, prospective observational studies, retrospective cohort studies, or multicenter registry-based investigations; (2) adult patients (≥18 years) who achieved return of spontaneous circulation (ROSC) after out-of-hospital cardiac arrest (OHCA) or in-hospital cardiac arrest (IHCA); (3) studies evaluating serum neuron-specific enolase (NSE) concentrations during post-resuscitation care; (4) studies reporting neurological outcomes, mortality, prognostic accuracy, or NSE threshold values; and (5) studies involving targeted temperature management (TTM), normothermia, or mixed post-cardiac arrest management strategies.

The exclusion criteria were as follows: (1) case reports, conference abstracts, editorials, letters, narrative reviews, systematic reviews, or meta-analyses; (2) animal or experimental studies; (3) pediatric-only populations; (4) studies without extractable outcome data; (5) duplicate publications or overlapping cohorts; and (6) studies evaluating neurological biomarkers unrelated to NSE without separate NSE data available for extraction.

When multiple studies originated from overlapping patient populations, the most comprehensive or methodologically robust study was included in the final analysis. Only studies reporting sufficient quantitative data for outcome assessment or meta-analysis were considered eligible.

### 2.3. Data Extraction and Quality Assessment

Two reviewers independently screened all retrieved studies and extracted relevant data using a standardized data collection form. Discrepancies during study selection, data extraction, or quality assessment were resolved through discussion and consensus, with involvement of a third reviewer when necessary.

The following variables were extracted from eligible studies: first author, year of publication, country, study design, sample size, patient demographics, type of cardiac arrest (OHCA/IHCA), initial cardiac rhythm, targeted temperature management (TTM) protocol, timing of NSE measurements, NSE concentrations and threshold values, neurological outcome assessment, mortality, follow-up duration, and reported measures of prognostic performance, including sensitivity, specificity, and area under the receiver operating characteristic curve (AUC).

The primary neurological outcome was assessed using the Cerebral Performance Category (CPC) [21] scale whenever available. Favorable neurological outcome was generally defined as CPC scores of 1–2, whereas poor neurological outcome corresponded to CPC scores of 3–5.

Risk of bias and methodological quality were independently evaluated by two reviewers using the Newcastle–Ottawa Scale (NOS) [22] for observational studies and the Cochrane Risk of Bias tool (RoB-2) [23] for randomized controlled trials, when applicable. The NOS evaluates three methodological domains, including selection of study groups, comparability of cohorts, and outcome assessment, with a maximum score of 9 points. Studies scoring 7–9 points were considered high quality, those scoring 5–6 points were considered moderate quality, and studies scoring fewer than 5 points were considered low quality.

The interpretation of the overall body of evidence was guided by the principles of the Grading of Recommendations Assessment, Development and Evaluation (GRADE) framework [24].

### 2.4. Outcome Measures and Definitions

The primary outcome of interest was poor neurological outcome after cardiac arrest, most commonly assessed using the Cerebral Performance Category (CPC) scale at hospital discharge or follow-up evaluation. Poor neurological outcome was generally defined as CPC scores of 3–5, whereas a favorable neurological outcome corresponded to CPC scores of 1–2.

Secondary outcomes included all-cause mortality, prognostic accuracy of serum neuron-specific enolase (NSE), optimal NSE threshold values for outcome prediction, sensitivity, specificity, area under the receiver operating characteristic curve (AUC), and the potential influence of targeted temperature management (TTM) on NSE kinetics and prognostic performance.

Additional outcome measures included comparisons between different temperature management strategies, serial NSE measurements at different time points following return of spontaneous circulation (ROSC), and multimodal prognostic approaches integrating NSE with electroencephalography or neuroimaging findings when available [5].

For studies reporting neurological outcomes at multiple time points, the longest available follow-up duration was preferentially considered for quantitative synthesis. When outcome definitions differed between studies, the original study definitions were recorded and harmonized whenever feasible for pooled analysis.

### 2.5. Statistical Analysis

Statistical analyses were performed using Review Manager (RevMan, version 5.4.1; Cochrane Collaboration, Copenhagen, Denmark) and R software (version 4.3.0; R Foundation for Statistical Computing, Vienna, Austria). Odds ratios (ORs) with 95% confidence intervals (CIs) were calculated for categorical variables, whereas continuous variables were analyzed using mean differences (MDs) or standardized mean differences (SMDs), as appropriate.

For studies reporting continuous variables as medians and ranges or interquartile ranges, data were converted to means and standard deviations using validated statistical methods when applicable [25].

Heterogeneity among studies was assessed using Cochran’s Q test and the I^2^ statistic. An I^2^ value > 50% or a *p*-value < 0.10 for Cochran’s Q test was considered indicative of substantial heterogeneity. Fixed-effects or random-effects models were selected according to the degree of heterogeneity. For the quantitative synthesis of AUC values, pooled estimates were calculated using an inverse-variance random-effects model based on the DerSimonian–Laird method, as implemented in Review Manager (RevMan, version 5.4.1). Individual AUC values and their corresponding 95% confidence intervals were extracted directly from the original publications. When standard errors were not explicitly reported, they were derived from the published 95% confidence intervals. No transformation of AUC values was performed prior to pooling. Pooled estimates were weighted using the inverse-variance method implemented in RevMan.

Subgroup analyses were planned according to the targeted temperature management strategy, the timing of NSE measurement, the neurological outcome definition, and the study design, as soon as sufficient data were available. Sensitivity analyses were performed by sequential exclusion of individual studies to evaluate the robustness and stability of the pooled estimates.

Publication bias was assessed by visual inspection of funnel plots. Formal statistical tests for funnel plot asymmetry, including Egger’s regression test, were planned but were not performed because fewer than ten studies were available for quantitative synthesis. Statistical significance was defined as a two-sided *p*-value < 0.05.

Where feasible, pooled sensitivity, specificity, and area under the receiver operating characteristic curve (AUC) values were additionally analyzed to evaluate the prognostic performance of NSE for neurological outcome prediction after cardiac arrest.

## 3. Results

### 3.1. Study Selection

The systematic literature search identified a total of 842 records across the PubMed/MEDLINE, Embase, and Scopus databases. After removal of duplicate records, 330 studies remained for title and abstract screening.

Following title and abstract screening, 305 records were excluded because they represented review articles, meta-analyses, editorials, conference abstracts without sufficient data, pediatric studies, animal investigations, or did not meet the predefined inclusion criteria. Consequently, 25 studies were considered potentially eligible and underwent full-text assessment.

After full-text review, four studies were excluded because of insufficient outcome data or lack of extractable quantitative NSE information. Ultimately, 21 studies met all eligibility criteria and were included in the qualitative synthesis. Of these, seven studies provided sufficiently homogeneous and extractable data for inclusion in the quantitative synthesis and meta-analysis.

**Figure 1:**
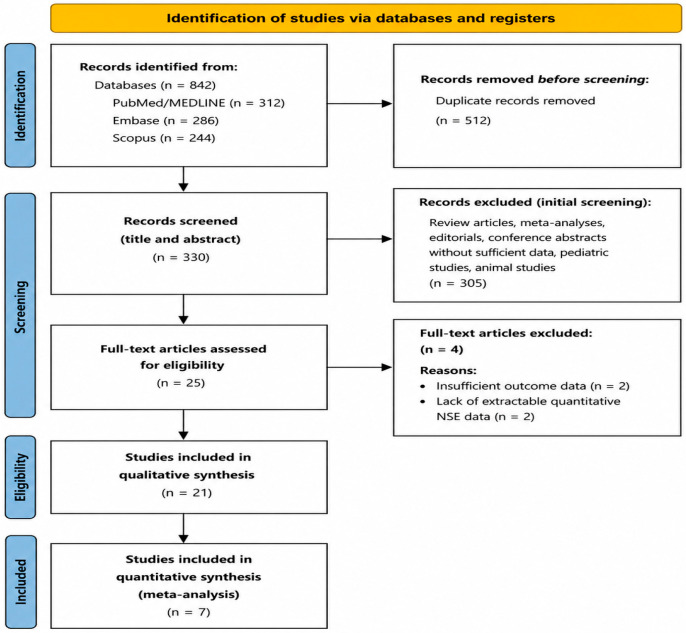
PRISMA 2020 flow diagram of the study selection process.

The study selection process was conducted in accordance with the PRISMA 2020 statement and is summarized in the PRISMA flow diagram (Figure 1).

### 3.2. Baseline Characteristics and Quality Assessment

A total of 21 studies met the predefined eligibility criteria and were included in the final qualitative synthesis. The included studies were published between 2009 and 2026 and predominantly consisted of prospective or retrospective observational cohort studies evaluating the prognostic role of neuron-specific enolase (NSE) in adult patients resuscitated after cardiac arrest. Several studies also represented post hoc analyses of randomized controlled trial cohorts and large multicenter registries.

The study populations primarily included patients with out-of-hospital cardiac arrest (OHCA), although several investigations also incorporated in-hospital cardiac arrest (IHCA) populations. Sample sizes varied substantially among studies, ranging from 31 patients in smaller pilot cohorts to more than 3300 participants in large multicenter analyses. Most studies enrolled comatose survivors after return of spontaneous circulation (ROSC) and evaluated neurological prognostication within the context of contemporary post-resuscitation care.

Targeted temperature management (TTM) was implemented in the majority of studies, with target temperatures generally ranging between 32 °C and 36 °C. NSE measurements were most commonly performed at 24, 48, and 72 h after ROSC, although several studies additionally assessed serial NSE kinetics or maximum NSE concentrations during the early post-resuscitation period.

Neurological outcomes were primarily assessed using the Cerebral Performance Category (CPC) scale or the modified Rankin Scale (mRS) at hospital discharge or during follow-up periods ranging from 3 to 12 months. Poor neurological outcome was defined as CPC 3–5 or mRS 4–6, while favorable neurological recovery corresponded to CPC 1–2 or mRS 0–3. Several studies additionally investigated long-term cognitive performance, favorable neurological recovery, or multimodal neuroprognostication strategies incorporating electroencephalography (EEG), somatosensory evoked potentials (SSEPs), neuroimaging, and additional biomarkers.

Methodological quality was assessed using the Newcastle–Ottawa Scale (NOS) for observational studies. Overall, the included studies demonstrated moderate-to-high methodological quality, with NOS scores ranging from 6 to 9 points. Nineteen studies were classified as high quality and two studies as moderate quality, while no study was rated as low quality. The principal sources of heterogeneity identified across studies included differences in NSE sampling protocols, variability in target temperature strategies, heterogeneity in reported prognostic thresholds, differences in follow-up duration, and potential confounding factors such as hemolysis and withdrawal-of-care decisions.

The baseline characteristics of the included studies are summarized in Table 1, while the methodological quality assessment is presented in Table 2.

### 3.3. Primary Outcome

The primary outcome of this systematic review and meta-analysis was the prognostic performance of serum neuron-specific enolase (NSE) for predicting neurological outcome after cardiac arrest. A total of 21 studies were included in the qualitative synthesis. However, only seven studies reported sufficiently homogeneous and extractable data on prognostic accuracy, expressed as area under the receiver operating characteristic curve (AUC), and were therefore eligible for quantitative synthesis.

Pooled analysis demonstrated that NSE exhibited good overall prognostic performance for neurological outcome prediction following cardiac arrest, with a pooled AUC of 0.868 (95% CI: 0.849–0.885). No significant between-study heterogeneity was observed (I^2^ = 0%). A sensitivity analysis restricted to studies evaluating poor neurological outcome as the primary endpoint yielded similar results, with a pooled AUC of 0.866 (95% CI: 0.845–0.884), confirming the robustness of the findings. Across the included studies, prognostic performance was generally highest when NSE was measured between 48 and 72 h after return of spontaneous circulation (ROSC). Reported AUC values ranged from approximately 0.79 to 0.90, indicating moderate-to-excellent predictive accuracy. Several studies additionally demonstrated that serial NSE kinetics improved prognostic discrimination compared with single measurements.

Overall, elevated NSE concentrations were consistently associated with unfavorable neurological outcomes, whereas lower NSE values were associated with favorable neurological recovery. These findings support the role of NSE as an important component of multimodal neuroprognostication after cardiac arrest.

**Figure 2:**
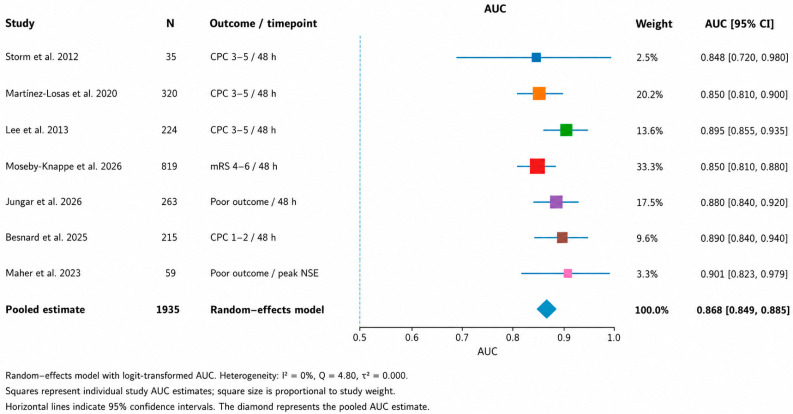
Forest plot of the prognostic accuracy of neuron-specific enolase (NSE) for predicting neurological outcome after cardiac arrest. Abbreviations: AUC, area under the receiver operating characteristic curve; CI, confidence interval; CPC, Cerebral Performance Category; mRS, modified Rankin Scale; NSE, neuron-specific enolase [16,19,31,32,34,37,41].

The pooled analysis of prognostic accuracy is presented in Figure 2.

The individual study-level AUC values included in the quantitative synthesis are provided in Supplementary Table S1.

The forest plot summarizes studies included in the quantitative synthesis evaluating the prognostic performance of NSE for neurological outcome prediction following cardiac arrest. Squares represent individual study estimates, with square size proportional to study weight in the random-effects model. Horizontal lines indicate 95% confidence intervals (CIs), and the diamond represents the pooled effect estimate.

Seven studies provided sufficiently homogeneous and extractable area under the receiver operating characteristic curve (AUC) data and were included in the meta-analysis. The pooled analysis demonstrated good overall prognostic accuracy of NSE, with a pooled AUC of 0.868 (95% CI: 0.849–0.885). No significant between-study heterogeneity was observed (I^2^ = 0%).

### 3.4. Secondary Outcome

#### 3.4.1. NSE Cut-Off Values for Poor Neurological Outcome

Considerable variability was observed regarding the optimal NSE threshold for predicting poor neurological outcome after cardiac arrest. Reported cut-off values ranged from approximately 28 μg/L to more than 100 μg/L, depending on sampling time, patient population, temperature management strategy, and acceptable false-positive rate.

Despite this variability, higher NSE concentrations were consistently associated with unfavorable neurological outcomes. Several studies identified thresholds with near-100% specificity for poor outcome prediction, although sensitivity varied substantially across cohorts. These findings suggest that NSE cut-off values should be interpreted within the clinical context and according to the timing of measurement.

#### 3.4.2. Influence of Targeted Temperature Management on NSE Performance

Most included studies evaluated NSE in patients treated with targeted temperature management (TTM). Evidence suggests that TTM may influence NSE kinetics and prognostic thresholds; however, NSE remained significantly associated with neurological outcome across different temperature management strategies.

Studies comparing hypothermia-treated and normothermic patients reported differences in optimal NSE cut-off values, whereas contemporary TTM and TTM2 cohorts confirmed the continued prognostic utility of NSE within modern post-resuscitation care.

#### 3.4.3. NSE Kinetics and Serial Measurements

Several studies assessed serial NSE measurements during the first 72 h after return of spontaneous circulation (ROSC). Overall, prognostic performance improved over time, with measurements obtained between 48 and 72 h generally demonstrating the highest predictive accuracy.

Studies evaluating NSE kinetics further suggested that serial measurements may provide greater prognostic value than isolated single-time-point assessments, particularly when integrated into multimodal neuroprognostication strategies.

#### 3.4.4. Multimodal Neuroprognostication

Recent investigations increasingly incorporated NSE into multimodal prognostic algorithms combining clinical examination, electroencephalography (EEG), neuroimaging, somatosensory evoked potentials (SSEPs), and additional biomarkers. These approaches consistently demonstrated superior prognostic performance compared with isolated biomarker assessment.

Overall, the available evidence supports the use of NSE as an important component of multimodal neuroprognostication rather than as a standalone predictor. Collectively, these findings indicate that NSE thresholds vary substantially across studies and clinical settings, whereas serial measurements obtained between 48 and 72 h after ROSC and interpreted within a multimodal framework appear to provide the most reliable prognostic information.

### 3.5. Sensitivity Analysis

A sensitivity analysis was performed to evaluate the robustness of the primary meta-analysis. Because one study primarily assessed a favorable neurological outcome rather than a poor neurological outcome, a secondary pooled analysis was conducted after excluding this study.

The results remained largely unchanged, with a pooled AUC of 0.866 (95% CI: 0.845–0.884) compared with 0.868 (95% CI: 0.849–0.885) in the primary analysis. No significant between-study heterogeneity was observed (I^2^ = 0%). Exclusion of the study by Besnard et al., which primarily evaluated a favorable neurological outcome, resulted in a pooled AUC of 0.866 (95% CI: 0.845–0.884; I^2^ = 0%), confirming the robustness of the findings.

These findings indicate that the overall results were stable and were not substantially influenced by the inclusion of individual studies with differing outcome definitions.

### 3.6. Publication Bias

**Figure 3:**
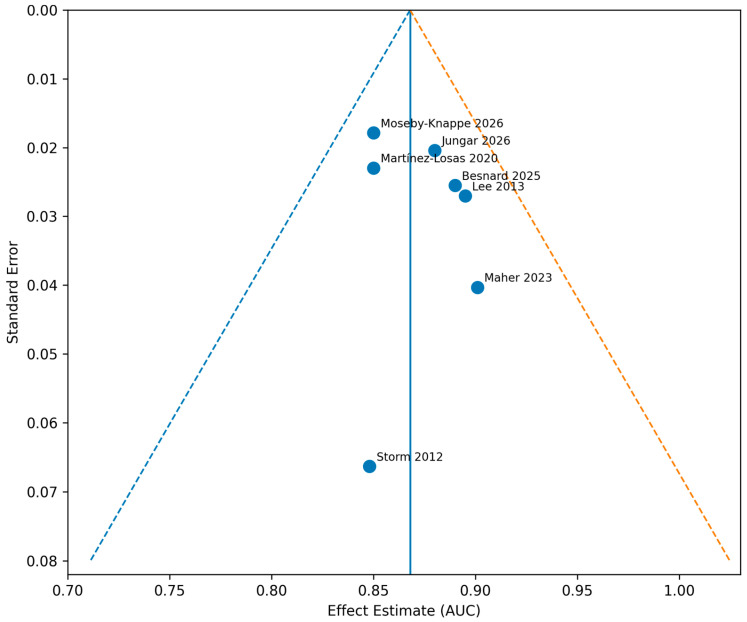
Funnel plot assessing potential publication bias among studies included in the meta-analysis. The funnel plot displays study-specific effect estimates (AUC values) against their standard errors. Visual inspection demonstrated an approximately symmetrical distribution of studies around the pooled effect estimate (AUC = 0.868), suggesting no substantial evidence of publication bias. Interpretation should be made with caution because only seven studies were available for quantitative synthesis [16,19,31,32,34,37,41].

Publication bias was assessed by visual inspection of a funnel plot (Figure 3). The distribution of studies appeared broadly symmetrical around the pooled effect estimate, with no obvious evidence of substantial small-study effects.

However, the interpretation of publication bias should be made with caution because only seven studies were available for quantitative synthesis. Current methodological recommendations indicate that formal statistical tests for funnel plot asymmetry have limited reliability when fewer than ten studies are included in a meta-analysis.

Overall, no clear evidence of significant publication bias was identified, although its presence cannot be completely excluded.

## 4. Discussion

### 4.1. Principal Findings

In this systematic review and meta-analysis, we evaluated the prognostic performance of neuron-specific enolase (NSE) for neurological outcome prediction after cardiac arrest, with particular emphasis on studies performed within contemporary post-resuscitation care frameworks. Twenty-one studies met the eligibility criteria for qualitative synthesis, while seven studies provided sufficiently homogeneous and extractable AUC data for quantitative analysis.

The principal finding of this study is that NSE demonstrated good overall prognostic accuracy for neurological outcome prediction following cardiac arrest. The pooled analysis yielded an AUC of 0.868 (95% CI: 0.849–0.885), with no significant between-study heterogeneity (I^2^ = 0%). These findings indicate consistent discriminatory performance across diverse populations and support the continued role of NSE as one of the most clinically relevant biomarkers of hypoxic–ischemic brain injury.

A second important observation was the substantial variability in reported NSE thresholds. Across the included studies, cut-off values associated with poor neurological outcome ranged from approximately 28 μg/L to more than 100 μg/L, depending on sampling time, assay methodology, patient characteristics, and temperature management strategy. This variability highlights the limitations of applying universal NSE thresholds in clinical practice.

Finally, the evidence synthesized in this review consistently supports the integration of NSE within multimodal neuroprognostication algorithms. Studies combining NSE with electroencephalography (EEG), somatosensory evoked potentials (SSEPs), neuroimaging findings, and clinical examination demonstrated superior prognostic performance compared with isolated biomarker assessment.

### 4.2. Comparison with Previous Literature

The findings of the present review are consistent with previous evidence identifying NSE as one of the most extensively validated biomarkers for neurological prognostication after cardiac arrest. Earlier studies established a clear relationship between increasing NSE concentrations and unfavorable neurological outcomes, leading to the incorporation of NSE into international post-resuscitation guidelines.

Our results are broadly aligned with previous systematic reviews and meta-analyses. Sharma et al. demonstrated that NSE possesses substantial prognostic value across a broad spectrum of cardiac arrest populations, while Kurek et al. confirmed its usefulness for predicting survival and neurological recovery after resuscitation. However, many earlier investigations included studies performed before the widespread adoption of contemporary temperature management strategies and modern multimodal prognostication pathways.

Although the pooled AUC observed in the present meta-analysis (0.868) appears slightly higher than those reported in previous systematic reviews, several factors may explain this difference. First, our quantitative synthesis included more recent studies reflecting contemporary post-resuscitation care and current targeted temperature management practices. Second, stricter eligibility criteria were applied for inclusion in the quantitative synthesis, resulting in a more homogeneous dataset. Third, only studies reporting directly comparable AUC estimates were included, thereby reducing methodological heterogeneity. Finally, differences in neurological outcome definitions, follow-up duration, and NSE sampling protocols across previous meta-analyses may also have contributed to the observed differences in pooled diagnostic performance.

An important strength of the present review is the inclusion of recent studies published between 2024 and 2026, including investigations derived from TTM and TTM2 populations. These contemporary cohorts provide evidence that remains directly applicable to current clinical practice and demonstrate that NSE retains significant prognostic value despite major changes in post-cardiac arrest management over the last decade.

Moreover, recent studies increasingly support individualized interpretation of NSE values rather than reliance on fixed thresholds. The growing emphasis on serial measurements, biomarker kinetics, and integration with electrophysiological findings reflects a transition from threshold-based prognostication toward personalized risk assessment.

### 4.3. Influence of Targeted Temperature Management

One of the central objectives of this review was to evaluate the relationship between NSE and targeted temperature management. Although TTM has become a cornerstone of post-resuscitation care, uncertainty remains regarding its influence on NSE release, metabolism, and prognostic thresholds.

Several studies included in this review reported differences in NSE kinetics between hypothermia-treated and normothermic patients. In particular, higher NSE thresholds were frequently required to achieve comparable specificity for poor neurological outcome in patients managed with TTM. These findings may partially explain the variability observed among published cut-off values and suggest that temperature management influences biomarker behavior.

Nevertheless, the overall prognostic utility of NSE remained preserved across different temperature management strategies. Contemporary studies, including analyses derived from TTM and TTM2 cohorts, consistently demonstrated significant associations between elevated NSE concentrations and unfavorable neurological outcomes. Therefore, the available evidence suggests that TTM modifies the interpretation of NSE values rather than eliminating their prognostic value.

From a clinical perspective, these findings support the use of NSE within modern temperature management protocols while emphasizing the importance of serial measurements and multimodal interpretation. Rigid application of historical NSE thresholds may no longer be appropriate in contemporary post-resuscitation care.

### 4.4. Clinical Implications

The results of this review reinforce current guideline recommendations that NSE should not be used as a standalone predictor of neurological outcome. Although the pooled AUC observed in the present meta-analysis indicates good prognostic performance, biomarker concentrations alone are insufficient to support irreversible clinical decisions.

Instead, NSE should be interpreted alongside neurological examination, EEG findings, SSEP results, neuroimaging studies, and other validated prognostic markers. Such an approach minimizes the risk of false-positive predictions and reflects current international recommendations for multimodal neuroprognostication.

The findings additionally support the routine use of serial NSE measurements between 48 and 72 h after return of spontaneous circulation. Several studies included in this review demonstrated improved prognostic accuracy at later time points, suggesting that temporal biomarker trajectories may provide greater clinical value than isolated single measurements.

### 4.5. Strengths and Limitations

This study has several strengths. A comprehensive search strategy was performed across multiple databases in accordance with PRISMA recommendations. Only studies involving adult cardiac arrest populations with reported NSE measurements and neurological outcomes were included. Furthermore, the review specifically evaluated the interaction between NSE and contemporary temperature management strategies, an area of increasing clinical importance.

Several limitations should also be acknowledged. First, although 21 studies were included in the qualitative synthesis, only seven provided sufficiently homogeneous and extractable AUC data for quantitative analysis. Second, heterogeneity existed regarding NSE sampling schedules, assay methodologies, temperature management protocols, and outcome definitions. Third, some studies reported outcomes using CPC scales, whereas others used modified Rankin Scale-based definitions. Finally, publication bias could not be assessed with high reliability because fewer than ten studies were available for quantitative synthesis.

### 4.6. Future Directions

Future investigations should focus on standardizing NSE measurement protocols, including assay methodology, timing of sampling, and clinically validated prognostic thresholds. Additional research evaluating serial NSE kinetics may further improve prognostic precision and facilitate the development of individualized prediction models.

The integration of NSE with emerging biomarkers such as neurofilament light chain (NfL) and glial fibrillary acidic protein (GFAP) represents another promising direction for future research. Ultimately, multimodal prognostic models combining biochemical, electrophysiological, and imaging parameters may provide more accurate and individualized neurological outcome prediction after cardiac arrest.

## 5. Conclusions

This systematic review and meta-analysis demonstrates that neuron-specific enolase (NSE) provides good prognostic accuracy for neurological outcome prediction after cardiac arrest, with a pooled AUC of 0.868. Elevated NSE concentrations were consistently associated with unfavorable neurological outcomes across diverse patient populations and temperature management strategies. However, substantial variability in reported prognostic thresholds highlights the need for cautious interpretation of NSE values in clinical practice.

The available evidence supports the use of serial NSE measurements, particularly between 48 and 72 h after return of spontaneous circulation, and reinforces current recommendations to integrate NSE within a multimodal neuroprognostication framework rather than using it as a standalone predictor. Future studies should focus on standardizing NSE measurement protocols, validating clinically relevant thresholds, and integrating NSE with emerging biomarkers to improve individualized neurological outcome prediction after cardiac arrest.

## Figures and Tables

**Table 1:** Characteristics of included studies.

Study	Year	Study Design	Population	Sample Size (*n*)	TTM	NSE Assessment	Neurological Outcome
Rundgren et al. [26]	2009	Prospective multicenter cohort	OHCA survivors	102	Yes	2, 24, 48, 72 h	CPC at 6 months
Steffen et al. [27]	2010	Prospective observational cohort	Cardiac arrest survivors	230	Mixed	72 h NSE	CPC at ICU discharge
Mörtberg et al. [28]	2011	Prospective observational study	OHCA survivors	31	Yes	Serial measurements	CPC at 6 months
Daubin et al. [29]	2011	Prospective cohort	Comatose CA survivors	97	Partial	24 h and 72 h	CPC at 3 months
Pfeifer et al. [30]	2011	Retrospective cohort	Cardiac arrest survivors	210	Mixed	NSE during ICU stay	Survival and neurological outcome
Storm et al. [31]	2012	Prospective cohort	Hypothermia-treated CA survivors	35	Yes	Daily NSE for 4 days	CPC at ICU discharge
Lee et al. [32]	2013	Retrospective cohort	TH-treated CA survivors	224	Yes	0, 24, 48 h	CPC at hospital discharge
Calderon et al. [33]	2014	Prospective cohort	Comatose CA survivors	72	Yes	0–72 h serial NSE	Survival to discharge
Martínez-Losas et al. [34]	2020	Retrospective cohort	TTM-treated CA survivors	320	Yes	Admission, 24, 48, 72 h	CPC at 3 months
Moseby-Knappe et al. [35]	2021	Post hoc TTM trial analysis	OHCA survivors	717	Yes	24, 48, 72 h	CPC at 6 months
Brønnick et al. [36]	2021	Prospective cohort	OHCA survivors with CPC ≤ 2	79	Yes	Arrival, 24, 48, 72 h	Cognitive outcome at 6 months
Kim et al. [10]	2023	Prospective multicenter registry	OHCA survivors treated with TTM	623	Yes	48 h NSE	CPC at 6 months
Maher et al. [37]	2023	Retrospective cohort	OOHCA survivors	59	Yes	Peak NSE	Long-term neurological outcome
Živanović et al. [38]	2024	Prospective observational study	Comatose CA survivors	86	Yes	NSE at 72 h	CPC outcome
Bougouin et al. [39]	2024	Prospective multicenter cohort	OHCA survivors	337	Mixed	NSE within ERC algorithm	mRS at 90 days
Lagebrant et al. [40]	2025	Post hoc multicenter analysis	OHCA survivors	3388	Mixed	NSE < 17 μg/L	Functional outcome at 6 months
Besnard et al. [41]	2025	Retrospective registry cohort	Comatose CA survivors	215	Mixed	24, 48, 72 h and kinetics	CPC at 3 months
Jungar et al. [16]	2026	Prospective cohort	Cardiac arrest biobank population	263	Mixed	48 h NSE	Neurological outcome
Kim et al. [42]	2026	Retrospective cohort	Comatose OHCA survivors	213	Yes	Maximum NSE < 72 h	Late awakening and CPC
Hong et al. [43]	2026	Retrospective cohort	OHCA survivors treated with TTM	124	Yes	0, 24, 48, 72 h	CPC at 6 months
Moseby-Knappe et al. [19]	2026	Prospective international observational study (TTM2)	OHCA survivors	819	Yes	0, 24, 48, 72 h	mRS at 6 months

Abbreviations: CA, cardiac arrest; CPC, Cerebral Performance Category; ICU, intensive care unit; mRS, modified Rankin Scale; NSE, neuron-specific enolase; OHCA, out-of-hospital cardiac arrest; OOHCA, out-of-hospital cardiac arrest; ROSC, return of spontaneous circulation; TH, therapeutic hypothermia; TTM, targeted temperature management.

**Table 2:** Risk of bias assessment of included studies using the Newcastle–Ottawa Scale.

Study	Selection	Comparability	Outcome	Total NOS Score	Quality
Rundgren et al., 2009 [26]	4	2	2	8/9	High
Steffen et al., 2010 [27]	3	1	3	7/9	High
Mörtberg et al., 2011 [28]	3	1	3	7/9	High
Daubin et al., 2011 [29]	4	2	2	8/9	High
Pfeifer et al., 2011 [30]	3	1	2	6/9	Moderate
Storm et al., 2012 [31]	3	1	3	7/9	High
Lee et al., 2013 [32]	3	1	3	7/9	High
Calderon et al., 2014 [33]	3	2	2	7/9	High
Martínez-Losas et al., 2020 [34]	3	1	3	7/9	High
Moseby-Knappe et al., 2021 [35]	4	2	2	8/9	High
Brønnick et al., 2021 [36]	3	1	3	7/9	High
Kim et al., 2023 [10]	4	2	3	9/9	High
Maher et al., 2023 [37]	3	1	2	6/9	Moderate
Živanović et al., 2024 [38]	3	1	3	7/9	High
Bougouin et al., 2024 [39]	4	2	3	9/9	High
Lagebrant et al., 2025 [40]	4	2	2	8/9	High
Besnard et al., 2025 [41]	3	2	3	8/9	High
Jungar et al., 2026 [16]	3	1	3	7/9	High
Kim et al., 2026 [42]	3	1	3	7/9	High
Hong et al., 2026 [43]	3	1	3	7/9	High
Moseby-Knappe et al., 2026 [19]	4	2	3	9/9	High

Abbreviations: NOS—Newcastle–Ottawa Scale. Selection was scored from 0 to 4 points, comparability from 0 to 2 points, and outcome assessment from 0 to 3 points. Studies were classified as high quality when scoring 7–9 points, moderate quality when scoring 5–6 points, and low quality when scoring fewer than 5 points.

## Data Availability

All data supporting the findings of this study are included within the article and its Supplementary Materials. The original datasets analyzed during the current study are available in the respective published articles cited in the References.

## Supplementary Materials

The following supporting information can be downloaded at: https://www.mdpi.com/article/10.3390/jcm15155798/s1, Supplementary File S1. PRISMA 2020 Checklist [20]; Supplementary Table S1. Individual studies included in the quantitative synthesis of the meta-analysis [16,19,31,32,34,37,41]; Supplementary Material S2. Complete database-specific search strategies.

## Abbreviations

The following abbreviations are used in this manuscript:

AUC	Area Under the Receiver Operating Characteristic Curve
CI	Confidence Interval
CPC	Cerebral Performance Category
EEG	Electroencephalography
GRADE	Grading of Recommendations Assessment, Development and Evaluation
IHCA	In-Hospital Cardiac Arrest
mRS	Modified Rankin Scale
NOS	Newcastle–Ottawa Scale
NSE	Neuron-Specific Enolase
OHCA	Out-of-Hospital Cardiac Arrest
PRISMA	Preferred Reporting Items for Systematic Reviews and Meta-Analyses
ROB	Risk of Bias
ROSC	Return of Spontaneous Circulation
SSEP	Somatosensory Evoked Potential
TTM	Targeted Temperature Management
