# Supplementary material for: Neuron-Specific Enolase for Neurological Prognostication After Cardiac Arrest in the Era of Targeted Temperature Management: A Systematic Review and Meta-Analysis
Source: J Clin Med. 2026 Jul 24;15(15):5798. doi: 10.3390/jcm15155798 (PMC13467247; doi:10.3390/jcm15155798)
Supplement: Supplementary file 1 [file jcm-15-05798-s001.zip › Supplementary Material S2.pdf]

Supplementary Table S1. Individual studies included in the quantitative synthesis of the meta-analysis [16,19,28,35,36,39,43].

**Study**	**N**	**AUC**	**95% CI**
Storm et al., 2012	35	0.848	0.728–0.940
Martínez-Losas et al., 2020	320	0.850	0.810–0.900
Lee et al., 2013	224	0.895	0.855–0.935
Moseby-Knappe et al., 2026	819	0.850	0.810–0.880
Jungar et al., 2026	263	0.880	0.840–0.920
Besnard et al., 2025	215	0.890	0.840–0.940
Maher et al., 2023	59	0.901	0.823–0.979

Abbreviations: AUC, area under the receiver operating characteristic curve; CI, confidence interval.

Supplementary Material S2. Complete database-specific search strategies.

A systematic literature search was conducted in PubMed/MEDLINE, Embase, and Scopus to identify studies evaluating the prognostic performance of neuron-specific enolase (NSE) after cardiac arrest. The search covered publications from January 2000 to April 2026. The final database search was performed on 30 April 2026. Searches were limited to English-language publications involving adult human subjects (≥18 years).

Database: PubMed/MEDLINE

Date of last search: 30 April 2026

Search strategy:

("Heart Arrest"[MeSH] OR "cardiac arrest" OR "out-of-hospital cardiac arrest" OR "in-hospital cardiac arrest" OR "post-cardiac arrest syndrome") AND ("Neuron-Specific Enolase"[MeSH] OR "neuron-specific enolase" OR NSE) AND ("Hypothermia, Induced"[MeSH] OR "targeted temperature management" OR "therapeutic hypothermia" OR "normothermia" OR "TTM") AND ("neuroprognostication" OR "prognosis" OR "biomarkers" OR "hypoxic-ischemic brain injury")

Filters applied

- Humans
- Adults (≥18 years)
- English language
- Publication period: January 2000–April 2026

Database: Embase

Date of last search: 30 April 2026

Search strategy:

("cardiac arrest"/exp OR "cardiac arrest" OR "out-of-hospital cardiac arrest" OR "in-hospital cardiac arrest" OR "post-cardiac arrest syndrome") AND ("neuron specific enolase"/exp OR "neuron specific enolase" OR "NSE") AND ("targeted temperature management" OR "therapeutic hypothermia"/exp OR "normothermia" OR "TTM") AND ("neuroprognostication" OR "prognosis" OR "biomarkers" OR "hypoxic ischemic brain injury")

Filters applied

- Humans
- Adults (≥18 years)
- English language
- Publication period: January 2000–April 2026

Database: Scopus

Date of last search: 30 April 2026

Search strategy:

TITLE-ABS-KEY

(("cardiac arrest" OR "out-of-hospital cardiac arrest" OR "in-hospital cardiac arrest" OR "post-cardiac arrest syndrome") AND ("neuron-specific enolase" OR "NSE") AND ("targeted temperature management" OR "therapeutic hypothermia" OR "normothermia" OR "TTM") AND ("neuroprognostication" OR "prognosis" OR "biomarkers" OR "hypoxic-ischemic brain injury"))

Filters applied

- Humans
- Adults (≥18 years)
- English language
- Publication period: January 2000–April 2026

Additional Search

The reference lists of all eligible studies and relevant review articles were manually screened to identify additional potentially eligible publications.
